# Number of top-quality embryos transferred has no advantage in women over the age of 40 in FET cycles

**DOI:** 10.1186/s12884-025-08075-0

**Published:** 2025-10-01

**Authors:** Shuai Liu, Li Yang, Hui Wang, Yunhao Liang, Huijiao Wu, Yu Jiang, Zhiheng Chen, Minna Yin

**Affiliations:** Center of Reproductive Medicine, Guangzhou Women and Children’s Medical Center, Guangzhou Medical University, Guangzhou, China

**Keywords:** Frozen thawed embryo transfer, Clinical pregnancy rate, Predictive factors, Top-quality embryos

## Abstract

**Introduction:**

The aim of the study was to explore the impact of the clinical and embryological factors on the pregnancy outcome of FET cycle, while determining the applicability range for the primary predictor.

**Methods:**

A total of 4395 FET cycles (2986 couples) were included in this retrospective study. A bootstrapping stepwise variable selection algorithm was used to identify independent predictors of the clinical pregnancy rate (CPR) from 24 clinical and embryological variables. Multivariate logistic regression was carried out to assess the impact of these predictors. The primary predictor was stratified to ascertain their applicability.

**Results:**

The final multivariate model incorporated the following 10 independent predictors: number of top-quality embryos transferred, age, number of in vitro fertilization/intracytoplasmic sperm injection attempts, endometrial thickness on trigger day, whether the embryo was cultured after thawing, fresh embryo transferred prior to FET, number of FET attempts, post-thaw embryo blastomere integrity, embryo developmental stage at transfer, and duration of infertility. The number of top-quality embryos transferred was found to be one of the most important predictors of pregnancy. For the younger age groups (≤ 30, 31–35 and 36–40 years), there was a significant increase in the CPR and live birth rate when more than one top-quality embryo was transferred. However, no significant differences were observed in the CPR between those with no or only one top quality embryo and those with two or more top-quality embryos transferred (14.0% vs. 18.3%, respectively; *p* = 0.553) in the group aged > 40 years.

**Conclusions:**

Our data reveal that the number of top-quality embryos transferred is one of the most important factors in predicting the pregnancy outcomes of FET. Further, the number of top-quality embryos transferred showed no benefit for women > 40 years in the FET cycles.

**Supplementary Information:**

The online version contains supplementary material available at 10.1186/s12884-025-08075-0.

## Introduction

Frozen-thawed embryo transfer (FET) is an important supplementary procedure in the treatment of infertility owing to its many benefits such as lowering the risk of ovarian hyperstimulation syndrome and increasing the use of elective single embryo transfer [1, 2]. Owing to the adoption of more efficient vitrification techniques, the utilization of freeze-thawed embryos has increased worldwide in the past decade because of their superior assisted reproductive technology outcomes [3, 4]. This has driven clinicians to improve the outcomes of FET cycles by carefully considering various FET characteristics.

Many studies have used multivariate models to identify the predictors of FET outcomes; however, there is still no consensus [5, 6]. Although numerous clinical and embryological factors affect FET outcomes, most previous studies [7–9] have analyzed only some of these factors. It is well-established that a higher maternal age at the time of freezing embryos leads to a worsening of pregnancy outcomes [10]. The embryo developmental stage at transfer and embryo grade are two key factors used to predict the clinical pregnancy rate (CPR) [11–13]. However, few studies have focused on factors such as blood or mucus in or on the transfer catheter after ET and fresh embryos transferred before FET. In addition, the impact of primary predictors on clinical outcomes has not been evaluated in these studies.

The aim of the present study was to identify independent clinical and embryological predictors of clinical pregnancy outcomes in FET cycles and ascertain the extent of applicability of the primary predictor.

## Methods

### Study design and population

We performed a retrospective, single-center cohort study on patients undergoing frozen-thawed embryo transfer from June 2010 and October 2019 at the Reproductive Medicine Center of Guangzhou Women and Children’s Medical Center. A total of 4395 FET cycles involving 2986 couples were enrolled. All included women were between the ages of 21 and 45 years, had a body mass index (BMI) greater than 17 kg/m² and less than 35 kg/m², and demonstrated a morphologically normal uterus on salpingogram and/or hysteroscopy. Women with any treatment elements involving preimplantation genetic testing, in vitro maturation, donor eggs or sperm, slow-freezing embryos were excluded from the study.

Based on a comprehensive literature review and our clinical experience, we identified 24 clinical and laboratory variables. A logistic regression analysis was performed to explore the relationship between these variables and CPR. To assess the impact of maternal age on the number of top-quality embryos transferred and pregnancy outcomes, all FET cycles were categorized into four age groups: ≤30, 31–35, 36–40, and > 40 years.

The study was approved by the Ethical Committee of Guangzhou Women and Children’s Medical Center, and Institutional Review Board approval was obtained (Approval Number: 2020-3).

### Protocols

Oocyte retrieval was performed 34–36 h after human chorionic gonadotropin administration, and fertilization was achieved using in vitro fertilization (IVF), intracytoplasmic sperm injection (ICSI), or 50% IVF + 50% ICSI in rare cases. Embryo grading was based on the appearance of the embryo(s) on day 3 before freezing. According to the Istanbul Consensus Workshop parameters [14, 15], embryos with a normal cleavage (7–9 even blastomeres), no multinucleation, and < 10% fragmentation were defined as top-quality embryos.

Embryo/blastocyst cryopreservation and warming protocols were performed using commercial kits. Cryopreservation was achieved by vitrification (JieYing Laboratory Inc., Canada) or a slow-freeze procedure (Irvine Scientific Inc., USA). The selection of cleavage- or blastocyst-stage embryos for ET depended on the availability of blastocyst-stage embryos, suggestions from clinicians, and patient choice.

### Outcome measurements

In this study, the primary endpoint was clinical pregnancy outcome in a completed FET treatment cycle, defined as the detection of a gestational sac with fetal heart pulsations on ultrasonography scanning 4–5 weeks following FET. The live birth rate (LBR) was defined as the delivery of a viable infant after 24 weeks of gestation. Miscarriage rate (MR) was defined as a spontaneous loss of pregnancy before 20 weeks of gestation. All other FET cycle outcomes were classified as nonpregnant.

### Statistical analysis

To probe differences in various clinical and laboratory parameters between the two patient groups (positive versus negative pregnancy outcome groups), categorical variables were compared using chi-square and Wilcoxon tests, whereas continuous variables were analyzed using Student’s t-test.

Multivariate logistic regression with a forward selection procedure was conducted to predict the individual impact of various factors on FET success. Those variables that had a greater clinical importance and larger variance were selected. Variance inflation factor was used to detect the multicollinearity with the criterion of less than 10. Receiver operating characteristic (ROC) curves were calculated to determine the relationship between the adjusted model and the CPR. The adjusted odds ratios (ORs) with the appropriate 95% confidence interval (CI) values and discriminatory accuracy, namely the of the ROC in the final regression model are shown in the Results section. SPSS software (version 25.0; SPSS Inc., Chicago, IL, USA) was used to analyze the results. A *P* < 0.05 was considered statistically significant.

## Results

### Baseline characteristics by pregnancy outcome

We evaluated 4395 FET cycles involving 2986 couples during the study period. A total of 2,130 (48.5%) clinical pregnancies were reported. The mean age of the patients was 32.4 ± 5.1 years.

The clinical and laboratory characteristics of the cohort are summarized in Tables 1 and 2, respectively. The CPR decreased significantly with increasing women age and duration of infertility (*P* < 0.001). Couples who achieved pregnancy had undergone fewer IVF/ICSI attempts than those who did not (1.2 ± 0.7 vs. 1.5 ± 1.0, *P* < 0.001). Similarly, those who conceived had fewer FET attempts compared with those who did not (1.3 ± 0.6 vs. 1.4 ± 0.7, *P* < 0.001). Additionally, endometrial thickness was significantly greater in patients who became pregnant compared to those who did not (9.8 ± 1.8 vs. 9.6 ± 1.7, *P* < 0.001).

**Table 1:** Characteristics on clinical parameters of the FET cycles by pregnancy outcome

Characteristics	Pregnant (*n* = 2130)	Not pregnant (*n* = 2265)	*P*-value
Age (year)	31.3 ± 4.4	33.3 ± 5.4	< 0.001^a^
BMI (kg/m2)	21.2 ± 2.8	21.2 ± 2.8	0.432^a^
Duration of infertility (year)	3.5 ± 2.7	4.0 ± 3.3	< 0.001^b^
Type of infertility			< 0.001^c^
Primary infertility	52.4 (1116)	45.0 (1019)	
Secondary infertility	47.6 (1014)	55.0 (1246)	
Indications			< 0.001^c^
Tubal factor	40.9 (872)	41.2 (934)	
Ovarian factor	5.5 (118)	6.8 (154)	
Endometriosis	3.7 (78)	3.6 (81)	
Male factor	20.3 (433)	16.6 (375)	
PGT	1.7 (36)	0.7 (16)	
Unexplained	1.1 (24)	0.7 (15)	
Other reasons	26.7 (569)	30.5 (690)	
Protocol			0.279^c^
Long protocol	63.7 (1357)	63.8 (1446)	
Short protocol	3.3 (71)	4.1 (93)	
GnRH antagonist protocol	20.0 (427)	19.0 (430)	
Luteal-phase ovulation	1.2 (26)	1.4 (32)	
Micro-stimulation	10.6 (225)	10.7 (242)	
PPOS	0.7 (14)	0.3 (6)	
Natural cycle	0.5 (10)	0.7 (16)	
No. of IVF/ICSI attempts	1.2 ± 0.7	1.5 ± 1.0	< 0.001^b^
No. of FET attempts	1.3 ± 0.6	1.4 ± 0.7	< 0.001^b^
Endometrial preparation			0.111^c^
HRT	58.5 (1245)	56.1 (1271)	
GnRHa-HRT	10.6 (225)	10.7 (242)	
Stimulated cycles	2.8 (59)	2.1 (48)	
Spontaneous	28.2 (601)	31.1 (704)	
Endometrial thickness (mm)	9.8 ± 1.8	9.6 ± 1.7	< 0.001^a^
Position of uterine			0.539^c^
Anteversion	84.1 (1791)	82.9 (1877)	
Retroposition	14.9 (317)	15.9 (361)	
Flat	1.0 (22)	1.2 (27)	
Catheter with blood after ET	9.2 (195)	10.8 (245)	0.067^c^
Catheter with mucus after ET			0.363^c^
None	86.5 (1842)	84.7 (1919)	
Less	11.1 (236)	12.8 (289)	
Moderate	0.5 (11)	0.6 (14)	
More	2.1 (41)	1.9 (43)	

BMI, body mass index; PGT, preimplantation genetic testing; GnRH, gonadotropin-releasing hormone; PPOS, progestin-primed ovarian stimulation; IVF, in vitro fertilization; ICSI, intracytoplasmic sperm injection; HRT, hormonal replacement therapy; GnRHa, gonadotropin-releasing hormone agonist

^a^Two-sample t-test

^b^Two-sample Wilcoxon test

^c^Pearson’s Chi-squared test

**Table 2:** Characteristics on laboratory parameters of the FET cycles by pregnancy outcome

Characteristics	Pregnant (*n* = 2130)	Not pregnant (*n* = 2265)	*P*-value
Fertilization method			0.013^c^
IVF	66.8 (1423)	70.7 (1602)	
ICSI	32.6 (694)	28.5 (646)	
IVF + ICSI	0.6 (13)	0.8 (17)	
M Ⅱ rate	81.5 ± 11.2	82.1 ± 11.3	0.102^a^
Fertilization rate	70.0 ± 17.4	71.1 ± 18.3	0.073^a^
Cleavage rate	97.5 ± 1.3	97.4 ± 1.4	0.934^a^
Top quality embryos formation rate	35.9 ± 22.9	34.9 ± 26.0	0.212^a^
Fresh embryo transferred prior to FET	20.9 (446)	29.2 (661)	< 0.001^c^
Embryo developmental stage at transfer			< 0.001^c^
Cleavage stage	81.4 (1733)	86.0 (1948)	
Blastocyst stage	15.1 (322)	10.2 (232)	
Cleavage + Blastocyst	3.5 (75)	3.8 (85)	
whether the embryo was cultured after thawing			< 0.001^c^
No-Cleavage stage	49.7 (1058)	44.3 (1004)	
No-Blastocyst stage	7.0 (149)	4.5 (101)	
No-Mixed	0.1 (2)	0.1 (2)	
Yes	43.2 (921)	51.1 (1158)	
No. of embryo transferred	2.0 ± 0.4	2.0 ± 0.5	0.169^b^
No. of top quality embryo transferred	1.0 ± 0.9	0.7 ± 0.8	< 0.001^b^
No. of embryo fully intact	93.0 (1981)	87.0 (1970)	< 0.001^c^

M Ⅱ: metaphase II; FET: frozen-thawed embryo transfer

^a^Two-sample t-test

^b^Two-sample Wilcoxon test

^c^Pearson’s Chi-squared test

Significant differences were observed between the negative and positive pregnancy outcome groups, including type of infertility, diagnosis of infertility, fresh embryo transferred prior to FET, embryo developmental stage at transfer, whether the embryo was cultured after thawing, number of top-quality embryos transferred, and post-thaw embryo with blastomere integrity (*P* < 0.05). No significant differences were found between the two groups for any of the other variables considered.

### Multivariate analysis of the risk factors of pregnancy

The multivariate logistic analyses revealed that female age, duration of infertility, number of IVF/ICSI/FET attempts, endometrial thickness on trigger day, fresh embryos transferred before FET, embryo developmental stage at transfer, whether the embryo was cultured after thawing, number of top-quality embryos transferred, and post-thaw embryos with blastomere integrity were significantly correlated with CPR (Table 3).

**Table 3:** Multivariable analysis of predictors in clinical pregnancy

Predictors	*P*-value	OR (95% CI)
Age (year)	< 0.001	
≤ 30	< 0.001	6.401 (4.477–9.150)
31–35	< 0.001	5.953 (4.179–8.480)
36–40	< 0.001	4.119 (2.867–5.919)
>40		Reference
Duration of infertility (year)	0.021	
≤ 3	0.021	1.168 (1.024–1.332)
>3		Reference
No. of IVF/ICSI attempts	< 0.001	
1	0.021	1.389 (1.050–1.837)
2	0.780	0.957 (0.702–1.304)
≥ 3		Reference
No. of FET attempts	0.002	
1	0.026	1.290 (0.986–1.689)
2	0.893	0.980 (0.735–1.308)
≥ 3		Reference
Endometrial thickness (mm) on trigger day	0.033	
≤ 7	0.010	0.582 (0.386–0.878)
7.1–12	0.345	0.896 (0.714–1.125)
> 12		Reference
Fresh embryo transferred prior to FET	< 0.001	
Transferred	< 0.001	0.710 (0.610–0.825)
Not transferred		Reference
Embryo developmental stage at transfer	< 0.001	
Cleavage stage	0.022	0.670 (0.475–0.945)
Blastocyst stage	0.199	1.303 (0.870–1.952)
Mixed		Reference
Whether to culture after thawing	0.014	
No-Cleavage stage	0.491	0.994 (0.695–1.422)
No-Blastocyst stage	0.628	1.290 (1.117–1.491)
No-Mixed	0.413	2.443 (0.265–22.483)
Yes		Reference
No. of top quality embryo transferred	< 0.001	1.481 (1.363–1.608)
Post-thaw embryo with integrity of blastomeres	0.049	
Integrity	0.049	1.179 (1.003–1.391)
Not Integrity		Reference

OR, odds ratio; CI, confidence interval

ROC analysis showed that the area under the curve (AUC) for the number of top-quality embryos transferred in FET cycles was 0.612 (95% CI: 0.593–0.629), which was higher than that for all other variables related to clinical pregnancy. The AUC for maternal age was 0.601 (95% CI: 0.584–0.617), representing the second-highest value (Table 4).

**Table 4:** Relative importance of predictors retained in the multivariable model

Predictors	*P*-value	AUC
No. of top quality embryo transferred	< 0.001	0.612 (0.593–0.629)
Age (year)	< 0.001	0.601 (0.584–0.617)
No. of IVF/ICSI attempts	< 0.001	0.548 (0.532–0.566)
Endometrial thickness (mm) on trigger day	< 0.001	0.544 (0.527–0.563)
Whether to culture after thawing	< 0.001	0.540 (0.522–0.556)
Fresh embryo transferred prior to FET	< 0.001	0.534 (0.517–0.550)
No. of FET attempts	0.001	0.530 (0.512–0.549)
Post-thaw embryo with integrity of blastomeres	0.003	0.529 (0.513–0.546)
Embryo developmental stage at transfer	0.010	0.522 (0.505–0.540)
Duration of infertility (year)	0.011	0.520 (0.501–0.533)
Final adjusted model	< 0.001	0.689 (0.669–0.703)

AUC, the area under the receiver operating characteristic curve

**Fig. 1:**
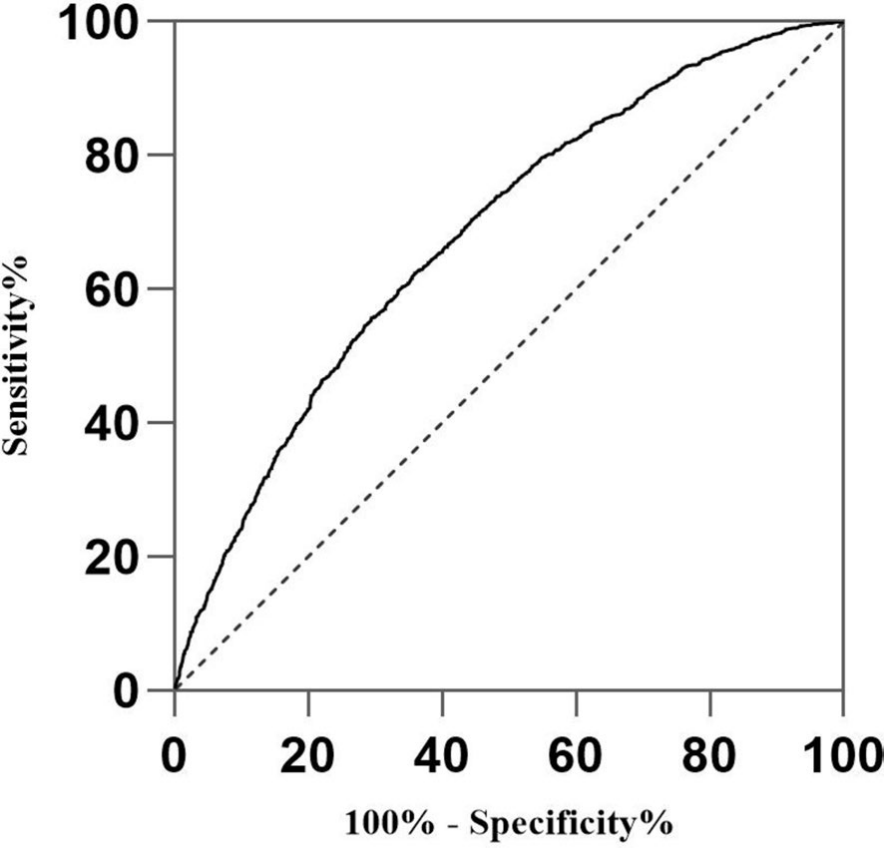
ROC curves of the adjust clinical pregnancy rate

After adjusting for the ten variables, an ROC curve was generated for the adjusted model to predict clinical pregnancy outcomes, with an AUC of 0.689 (95% CI: 0.669–0.703) (Fig. 1).

### Maternal age on the number of top-quality embryos transferred and pregnancy outcomes

For the younger age groups (≤ 30, 31–35 and 36–40 years), the CPR showed significant differences between those with no or only one top-quality embryo transferred and those with two or more top-quality embryos transferred (all *P* < 0.001). However, among women with advanced age (> 40 years), there were no significant differences in CPR between those with one and those with two or more top-quality embryos transferred (14.0% vs. 18.3%, *P* = 0.396) (Table 5).

**Table 5:** Clinical pregnancy rate and AUC of number of top quality embryos transferred within different age strata

Age	No. of top quality embryo transferred				
	≤ 1	≥ 2	*P*-value	AUC	*P*-value
≤ 30	593/1192 (49.7)	392/581 (67.5)	< 0.001	0.611 (0.583– 0.635)	< 0.001
31–35	501/1112 (45.1)	283/429 (66.0)	< 0.001	0.620 (0.592– 0.650)	< 0.001
36–40	213/585 (36.4)	102/186 (54.8)	< 0.001	0.596 (0.553– 0.637)	< 0.001
> 40	35/250 (14.0)	11/60 (18.3)	0.396	0.527 (0.434– 0.619)	0.562

**Fig. 2:**
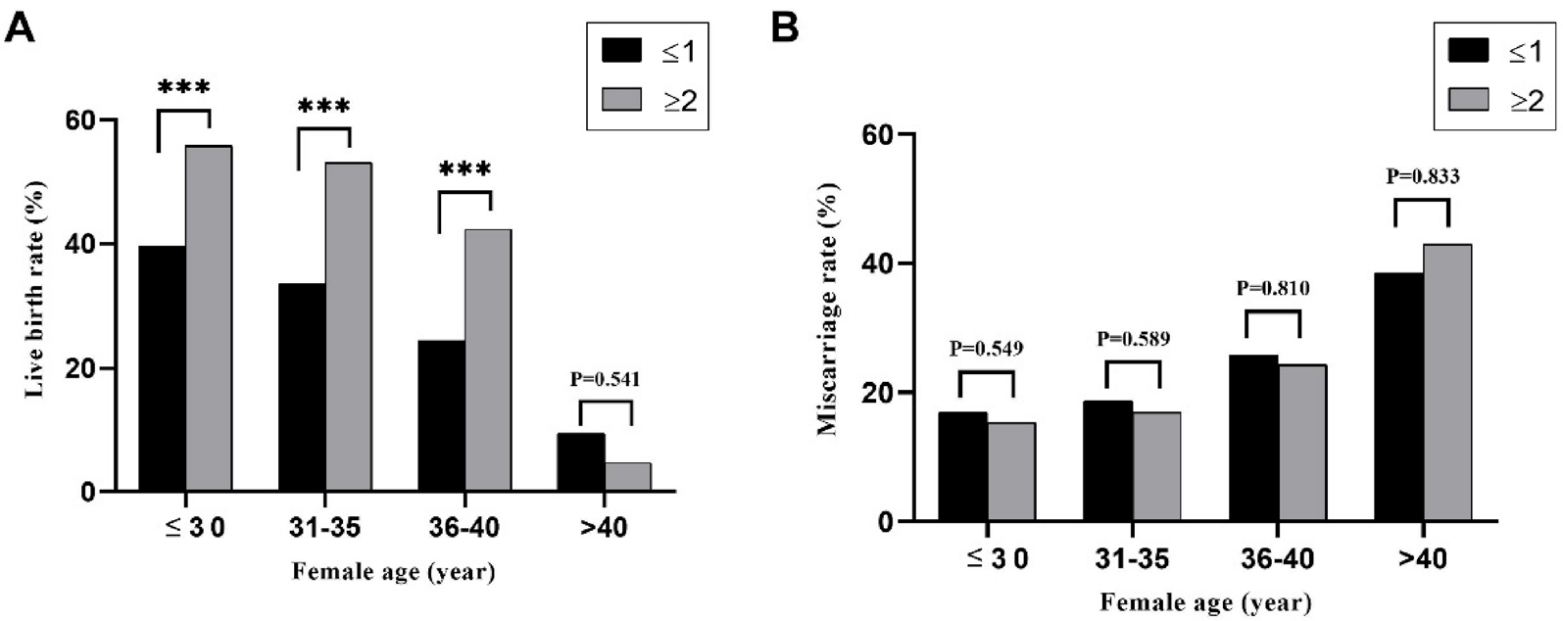
The correlation between the number of top-quality embryos transferred and the rates of live birth and miscarriage. (A) The live birth rate of the number of top- quality embryos transferred within different age strata (≤ 30, 31–35, 36–40, > 40 years). (B) The miscarriage rate of number of top-quality embryos transferred within different age strata. Black bars represent the number of top-quality embryos transferred ≤ 1; Grey bars represent the number of top-quality embryos transferred ≥ 2

Similarly, The LBR increased significantly with increasing number of top-quality embryos transferred in the groups with maternal ages ≤ 30, 31–35, and 36–40 years (39.4% vs. 55.8%, *P* < 0.001; 33.3% vs. 53.2%, *P* < 0.001; and 24.4% vs. 42.5%, *P* < 0.001). However, the > 40- year maternal age group showed no significant differences in live births with the increasing number of top-quality embryos transferred (Fig. 2A). Additionally, miscarriage rates showed no significant change with the number of top-quality embryos transferred in the different age strata (Fig. 2B). The distribution of top-quality embryos transferred did not differ among the various maternal age groups (Supplementary Fig. 1).

## Discussion

In this retrospective study, we focused on embryo cryopreservation and assessed the various clinical and embryological factors that affect pregnancy outcomes after FET cycles. Among the parameters analyzed, the number of top-quality embryos transferred was one of the most important determinants for improving the chances of clinical pregnancy after FET. Further, the number of top-quality embryos transferred showed no benefit for women > 40 years in the FET cycles.

The criteria impacting the quality of embryos include several variables that have previously been reported to affect FET-derived pregnancy outcomes, namely maternal age [10, 16], endometrial thickness on trigger day [17, 18], high-quality embryos [12, 19], post-thaw embryos with blastomere integrity [6, 8], and whether the embryo was cultured after thawing [6, 9]. This study validates these factors. Other predictors of pregnancy outcome included the number of top-quality embryos transferred, number of IVF/ICSI attempts, fresh embryos transferred prior to FET, number of FET attempts, embryo developmental stage at transfer, and number of embryos transferred.

Embryo quality is most commonly assessed by morphological evaluation. High-quality embryos are likely to exhibit better cryopreservation, survival, and implantation rates [20]. The presence of morphologically robust frozen embryos was associated with a higher CPR. In our study, a consistently high CPR and LBR as well as a decreased likelihood of miscarriage after FET in the younger maternal age groups (≤ 30, 31–35, and 36–40 years) implies that these groups of patients will benefit from increasing the number of top-quality embryos transferred.

It is known that IVF-derived pregnancy outcomes are associated with maternal age [21]. The present study demonstrated that age was the second most important predictor of clinical pregnancy outcomes in FET cycles. Therefore, it is reasonable and important to control for the impact of maternal age when the relationship between the number of top-quality embryos transferred and CPR is assessed. Therefore, we divided the patients into young and advanced maternal age groups. Among women with two or more top-quality embryos transferred, the likelihood of pregnancy increased in the younger maternal age group, and a high proportion achieved pregnancy. Conversely, the CPRs in the advanced maternal age group were quite low (< 20%), regardless of the number of top-quality embryos transferred (Table 5).

The number of top-quality embryos the most important predictor among younger maternal age groups and its predictive value declined among women aged > 40 years. A possible explanation is that advanced maternal age is associated with decreased uterine receptivity that can reduce the likelihood of successful embryo implantation [22]. This decline in uterine receptivity may diminish the effectiveness of top-quality embryos in achieving successful pregnancies in older women [23].

In addition, the risk of embryonic genetic errors increases with advancing maternal age [24–26]. Minasi et al. [27] reported an increasing probability of aneuploidy with a 10% per year increase in maternal age. Fragouli et al. [28] have reported that aneuploidy is as high as 17% in patients ≥ 40 years. The euploidy rates of embryos displaying robust morphology and development also increased from 21% in patients aged > 40 years to 44% in patients aged < 35 years [29].

Moreover, good embryo morphology scores appeared to be better correlated with the euploidy status of blastocysts than with cleavage embryos [30, 31]. This may be due to embryo genome activation that begins at the four- to eight-cell stage. Consequently, after the third day of culture, the embryonic genome begins to take control, and any genetic abnormalities will affect the embryo [32]. In our clinical unit, 83.9% of the patients chose to freeze embryos at the cleavage stage rather than at the blastocyst stage.

First, the retrospective nature of this study may have been a source of bias. Women aged > 40 years had undergone a greater number of prior IVF cycles than women in the younger maternal age groups, and the second attempt was of lower quality. Specifically, the lack of distinction between embryos frozen in a ‘‘freeze-all’’ policy and transfer of top-quality embryos in a fresh cycle was a limitation of this study. Additionally, the genetic status of the embryos had not been investigated. However, to overcome the lack of randomization between the maternal age groups, a comprehensive analysis revealed several possible confounding factors affecting FET outcomes.

## Conclusions

The number of top-quality embryos transferred was one of the most important predictors of pregnancy outcome in FET. Additionally, among women aged > 40 years, there was no difference in the CPR, LBR, or MR between those with one and those with two or more top-quality embryos transferred. As the likelihood of a pregnancy in women aged > 40 years from the transfer of one top-quality embryo was the same as that from the transfer of two or more top-quality embryos, women of advanced maternal age were proposed to undergo FET regardless of the number of top-quality embryos transferred.

## Supplementary Information

Below is the link to the electronic supplementary material.

**Supplementary Material 1: Supplementary Fig. 1** The distribution of top-quality embryos transferred across four age groups of females (≤30, 31–35, 36–40, >40 years).

## Data Availability

The datasets used and analyzed during the current study are available from the corresponding author on reasonable request.

## Abbreviations

FET: Frozen thawed embryo transfer
CPR: Clinical pregnancy rate
BMI: Body mass index
LBR: Live birth rate
IVF: In vitro fertilization
ICSI: Intracytoplasmic sperm injection
ROC: Receiver operating characteristic
OR: Odds ratio
CI: Confidence interval
AUC: Area under the curve
