# Supplementary figures and images for: Number of top-quality embryos transferred has no advantage in women over the age of 40 in FET cycles
Source: BMC Pregnancy Childbirth. 2025 Oct 1;25:984. doi: 10.1186/s12884-025-08075-0 (PMC12486488; doi:10.1186/s12884-025-08075-0)

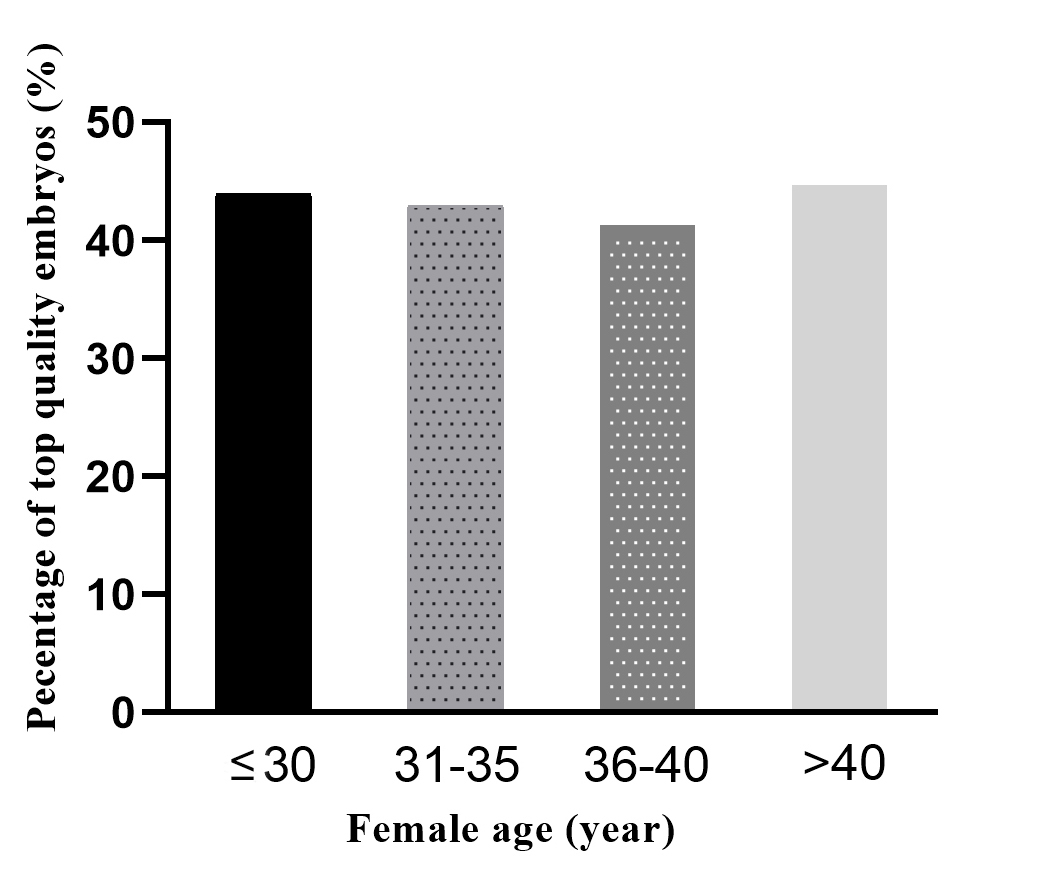

Supplement: Supplementary file 1 — Supplementary Material 1: Supplementary Fig. 1 The distribution of top-quality embryos transferred across four age groups of females (≤30, 31–35, 36–40, >40 years). [file 12884_2025_8075_MOESM1_ESM.jpg]
